# Classification of Potential Water Bodies Using Landsat 8 OLI and a Combination of Two Boosted Random Forest Classifiers

**DOI:** 10.3390/s150613763

**Published:** 2015-06-11

**Authors:** Byoung Chul Ko, Hyeong Hun Kim, Jae Yeal Nam

**Affiliations:** Department of Computer Engineering, Keimyung University, Sindang-dong, Dalseo-gu, Daegu 704-701, Korea; E-Mails: h2k@kmu.ac.kr (H.H.K.); jynam@kmu.ac.kr (J.Y.N.)

**Keywords:** Landsat 8, OLI sensor, water body classification, boosted random forest

## Abstract

This study proposes a new water body classification method using top-of-atmosphere (TOA) reflectance and water indices (WIs) of the Landsat 8 Operational Land Imager (OLI) sensor and its corresponding random forest classifiers. In this study, multispectral images from the OLI sensor are represented as TOA reflectance and WI values because a classification result using two measures is better than raw spectral images. Two types of boosted random forest (BRF) classifiers are learned using TOA reflectance and WI values, respectively, instead of the heuristic threshold or unsupervised methods. The final probability is summed linearly using the probabilities of two different BRFs to classify image pixels to water class. This study first demonstrates that the Landsat 8 OLI sensor has higher classification rate because it provides improved signal-to-ratio radiometric by using 12-bit quantization of the data instead of 8-bit as available from other sensors. In addition, we prove that the performance of the proposed combination of two BRF classifiers shows robust water body classification results, regardless of topology, river properties, and background environment.

## 1. Introduction

Maintaining clean rivers and lakes is a prerequisite for supplying stable and safe water for humans. Conventional water quality assessments are limited to *in situ* collection and measurement of water samples from several spots of a long river or a wide lake for subsequent laboratory analyses [1]. Even though this method is accurate, it requires substantial time and effort for continuous observation; therefore, satellite remote sensing has been used because of its cost-effectiveness and ability to overcome the constraints of conventional methods. Satellite remote sensing gathers water quality information over a greater range of temporal and spatial scales [1,2].

Among several available satellite remote sensors, the Thematic Mapper (TM) and Enhanced Thematic Mapper Plus (ETM+) sensors provided by the Landsat 5 and 7 satellites, respectively, are widely used for water quality assessment [3]. Landsat satellites have moderate spatial resolution (30 m), multi-spectral images (seven or eight bands), and a short revisit interval (16 days) [4]. Landsat 8, launched on 11 February 2013, carries an improved Operational Land Imager (OLI) sensor and the Thermal InfraRed Sensor (TIRS). The OLI sensor provides nine spectral bands (1~9) and TIRS provides two spectral bands (10~11), as shown in Table 1. Seven bands from band 2 to band 7 of OLI are consistent with the TM and ETM+ sensors. The new two spectral bands, band 1 and band 9 allows measuring water resources and coastal zone investigation and improving the detection of cirrus clouds. TIRS conducts thermal imaging can be applicable to evapotranspiration rate measure for water management [5].

**Table 1:** Wavelength range and spatial resolution of the Landsat 8 OLI and TIRS [5,6].

Band	Wavelength Range (μm)	Spatial Resolution (m)
OLI 1	0.433~0.453 (coastal/aerosol)	30
OLI 2	0.450~0.515 (blue)	30
OLI 3	0.525~0.600 (green)	30
OLI 4	0.630~0.680 (red)	30
OLI 5	0.845~0.885 (Near-IR)	30
OLI 6	1.560~1.660 (SWIR-1)	30
OLI 7	2.100~2.300 (SWIR-2)	30
OLI 8	0.500~0.680 (Pan)	15
OLI 9	1.360~1.390 (Cirrus)	30
TIRS 10	10.30~11.30 (LWIR-1)	100
TIRS 11	11.50~12.50 (LWIR-2)	100

The OLI sensor provides better signal to noise ratio (SNR) radiometric performance than other sensors because it uses 12-bit quantization of the data. Improved SNR performance means more bits are available for better land cover characterization [5]. In addition, OLI’s higher SNR makes it possible to narrow the spectral bands and reduce the sensitivity of the changes in the atmosphere [7].

In general, multispectral images obtained through satellite remote sensing have different spectral variations according to the land cover types, such as seawater, vegetation, urban areas, and mountain regions. Therefore, water body classification is the first step to assess the water quality automatically. Conventional water body classification methods apply one or more heuristic thresholds to spectral images. These methods are simple and obtain good classification results from limited terrain. However, these methods result in several false classifications when images consist of complex topologies such as mountain shadows, roads, and urban areas as well as rivers and lakes. Recently, Jiang *et al.* [4] proposed automatic river and lake extraction methods by applying the heuristic threshold method. This method combined the water indices (WIs) with sequences of thresholds that were determined by experiments to extract wide rivers and narrow rivers separately. However, this method has the same problems as threshold-based methods according to the artificial or natural terrain change.

Classifier-based methods deliver better water body classification performance than threshold-based methods because these methods do not need to set heuristic thresholds. In these types of methods, supervised and non-supervised learning techniques are used for water body classification with multispectral images. As for the supervised learning, neural networks [8] and support vector machine (SVM) [9] are representative classification methods. In the case of unsupervised learning, region growing [10] and ISODATA clustering [2] methods are frequently used in water body classification. Even though the two approaches produce better classification results than threshold-based methods, they still have two disadvantages. First, supervised learning needs expert experience or existing reference data to select appropriate training data [4]. In particular, even though the SVM classifier is a reasonable choice for general classification due to its high performance and accuracy, it is not suitable when the feature has high-dimensionality and the test data is over 1000 dimensions, due to computational complexity [11]. In contrast, random forest (RF) classifier that is an ensemble of decision trees has been shown to be effective in a large variety of high-dimensional problems, with high computational performance and accuracy than other supervised classifiers [11]. Second, unsupervised learning methods need additional post processing to merge particle regions into real rivers and lakes.

### Contributions of This Work

**Figure 1:**
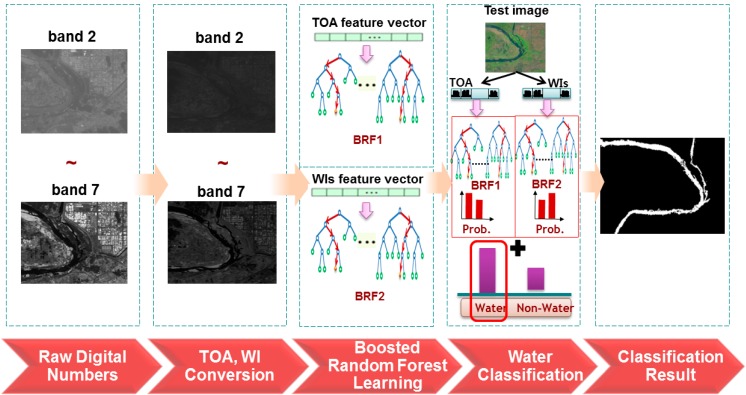
The block diagram of the water body classification procedure using OLI sensor data.

To solve the problem of supervised learning, our study proposes a new water body classification algorithm that uses a combination of two boosted random forest (BRF) classifiers based on top-of-atmosphere (TOA) reflectance values and spectral WIs, which were estimated only from the Landsat 8 OLI sensors without using TIRS. Figure 1 shows the block diagram of the water body classification procedure using OLI sensor data. In the second stage, multispectral images from the OLI sensor are represented as TOA reflectance and WI values because a classification result using two measures is better than raw spectral images. In the third stage, two types of BRF classifiers are learned using TOA reflectance and WI values of training data instead of the heuristic threshold or unsupervised methods. The learned BRF classifiers are used to detect the most likely water pixels in the test image in the fourth stage.

This study demonstrates the robust water body classification results of the proposed method by comparing them with spectral images of other Landsat series and state-of-the-art water body classification methods.

The remainder of this paper is organized as follows: in Section 2, the image conversion method to TOA reflectance and WIs is described. In Section 3, the proposed water body classification method using two types of BRF is introduced. In Section 4, we present experiments demonstrating the accuracy of our proposed classification method. Finally, our conclusions and scope for future work are presented in Section 5.

## 2. Conversion to TOA Reflectance and WIs

For a water body classification method with higher accuracy, we use two parameters, *i.e.*, TOA reflectance and WIs. Originally, the raw digital number (DN) of each spectral band is in a 16-bit unsigned integer format and can be rescaled to the TOA reflectance using radiometric rescaling coefficients provided in the product metadata file [5]. TOA reflectance has the two following advantages when compared with raw DN [3]:

- it removes the cosine effect at different solar zenith angles due to time difference between data acquisitions;
- it compensates for different values of the exoatmospheric solar irradiance.

In addition, the WIs are designed to enhance the classification performance between water bodies and land [4].

### 2.1. Conversion to TOA Reflectance

TOA reflectance can be calculated using OLI band data from the reflectance rescaling coefficients provided in the product metadata file. Conversion of the DN of OLI data to TOA reflectance (${\rho^{\prime }}_{\lambda }$), without correction for solar angle, is performed using the following formula [5]:

(1) $${\rho^{\prime }}_{\lambda }=M_{p}Q_{cal}+A_{p}$$

where $M_{p}$ and $A_{p}$ are the band-specific multiplicative and additive rescaling factors from the metadata, respectively. $Q_{cal}$ is the quantized and calibrated standard product pixel values. ${\rho^{\prime }}_{\lambda }$ does not contain a correction for the Sun angle; hence, the TOA reflectance value with a correction ($\rho_{\lambda }$) for the Sun angle is computed by:

(2) $$\rho_{\lambda }=\frac{{\rho^{\prime }}_{\lambda }}{\cos(\theta_{SZ})}=\frac{{\rho^{\prime }}_{\lambda }}{\sin(\theta_{SE})}$$

where $\theta_{SE}$ is the local Sun elevation angle provided in the metadata and $\theta_{SZ}$ is the local solar zenith angle estimated by $\left(90{}^{\circ}-\theta_{SE}\right)$. This study computes only six TOA reflectance values from band 2 to 7 except for band 1, 8, and 9 because of their specific purpose; Band 1 is used for investigating ocean colour and band 8 works just like panchromatic film instead of collecting visible colours. Band 9 is used for detecting cirrus contamination in other bands.

### 2.2. Water Index Estimation

As the second feature, we use normalized-difference water index (NDWI) [12] and modified NDWI (MNDWI) [13] because they have been successfully used in several water body classification methods [3,4,12,13]. NDWI is designed to maximize the reflectance of a water body by using green wavelength, minimize the low reflectance in Near-IR, and take advantage of the high reflectance in Near-IR of vegetable and soil features [3]. Xu’s MNDWI [13] was developed to enhance open water features by modifying NDWI. Moreover it can efficiently suppress and even remove built-up land noise as well as vegetation and soil noise. For estimating NDWI and MNDWI, this study used TOA reflectance as the same method of [3,14,15]:

(3) $$NDWI=(\rho_{3}-\rho_{5})/(\rho_{3}+\rho_{5})$$

(4) $$MNDWI_{36}=(\rho_{3}-\rho_{6})/(\rho_{3}+\rho_{6})$$

(5) $$MNDWI_{37}=(\rho_{3}-\rho_{7})/(\rho_{3}+\rho_{7})$$

where the subscript of ρ represents the TOA reflectance value computed from band 2 (blue), band 3 (green), band 5 (Near-IR), band 6 (SWIR-1), and band 7 (SWIR-2) of the Landsat 8 OLI wavelength.

## 3. Water Body Classification Using Combination of Boosted Random Forest (BRF)

For water body classification, we first remove shadow pixels using the relation ρ_3_ < *T_shadow_* because band 3 (green) is a more distinguishable band for differentiating water bodies from mountain or hill shadows than other bands [16]. Here, $T_{shadow}$ are the control parameters, large values of which can remove real water bodies, whereas small values generate wide false water bodies. This paper sets the initial values of $T_{shadow}$ as 0.08 based on several experiments.

After removing shadow pixels, we use a BRF classifier that is an ensemble of boosted randomized decision trees to classify water bodies accurately. Even though the random forest (RF) classifier [17] requires existing reference data in the training process like other supervised learning methods, the RF classifier is known to be effective for a large variety of high-dimensional problems with higher computational performance and accuracy than other classifiers, such as SVM or neural networks [11]. In addition, because Landsat images have large resolution, RF is more efficient method than other classifier in terms of processing speed and accuracy. However, it depends heavily on the number of decision trees and requires a certain amount of memory and CPU capacity. Therefore, BRF [18] is applied to our classification system to maintain the generality with a small number of decision trees when considering the fact that sequential training constructs complementary decision trees for the training samples.

In this study, two types of BRF classifiers are learned separately using different feature vectors instead of aggregating as one feature according to the experimental results of [19]. In reference [19], the author proved that if the basic characteristics of the two features were different, an artificial combination of two different features may worsen the classification performance. In particular, the performance of a random forest, which was the classifier used in this study, can be improved when the random forest uses the same types of feature. The first BRF classifier is trained using TOA reflectance values computed from six bands. The second BRF classifier is trained using three WIs computed from TOA reflectance values. For the training of an individual BRF, training data are constructed by the user. This training data include rivers and lakes as positive data and urban and lands as negative data. Then, six types of TOA reflectance values are extracted from positive and negative data for training the first BRF. For training the second BRF, three WIs features are extracted from the same positive and negative data. To perform the training, 7500 image pixels were randomly selected (2500 pixels from water bodies, 2500 pixels from urban regions, and 2500 pixels from mountains). The training data is collected from Seoul City and included the urban region and Hangang River and Daegu City that included the Palgong Mountain area. Form each training pixel, TOA and WI feature vectors are extracted and these features are applied to BRFs for classifier training. The comparative methods, RF classifier and SVM are also used the same training data and the performance comparison is described in Section 4.

Here, we construct two BRFs, *BRF*_1_ and *BRF*_2_ for each pixel: one uses only the TOA feature and the other uses only the WI feature. Because the basic characteristics of the TOA and WIs are different, we create two different BRFs rather than combining these into one feature vector according to the experiments of Ko *et al.* [19]. BRF adds a bootstrapping phase during the learning step, which is similar to the Adaboost algorithm. The learning of the BRF is summarized below (Algorithm 1).

**Algorithm 1** BRF learning1. *T*: the maximum number of decision trees to grow for BRF  *D*: the maximum depth of trees to extend M: number of classes *S_n_*: Training set, including positive (river and lake) and negative (land, mountain and building) samples with their labels and weight, {**x**_1_, *y*_1_, *w*_1_},…,{**x***_N_*, *y_N_*, *w_N_*}; **x***_i_* ∈ *X*, *y* ∈ *M* Initialize sample weight $w_{i}^{\mathrm{(1)}}$ = 1/*N*2. **For**
*t* = 1 to *T*
**do** Select subset *s* from training set *S_n_* Grow an unpruned tree using the *s* subset samples with their corresponding weights.  **For**
*d* = 1 to *D*
**do**  Each internal node randomly selects *p* variables and determines the best split function using only these variables.  **Loop:** Using different *p*-th variables, the split function *f*(*v_p_*) iteratively splits the training data into left (*Il*) and right (*Ir*) subsets using Equation (6).
(6)$$\begin{array}{l} Il=\{p\in In|f(vp)<t\},\\ Ir=In\backslash Il \end{array}$$
  The threshold *t* is randomly chosen by the split function *f*(*v_p_*) in the range $t\in (\underset{p}{\min}f(v_{p}),\underset{p}{\max}f(v_{p}))$.  Compute information gain Δ*G* function *f*(*v_p_*)  **If** (Δ*G*= max) **then** Determine the best split function *f*(*v_p_*) for the node *d*    **Else** goto Loop.  **End For** Store the probability distribution *P* (*C* | *l_t_*) to leaf node Output: A weak decision treeEstimate class label ${\hat{y}}_{i}$ of the training data with the trained decision trees:
(7)${\hat{y}}_{i}=\arg\text{​}\underset{c}{\max}P(c|l_{t})$ Calculate the error of decision tree *ε_t_*:
(8)$\varepsilon_{t}=\sum_{i:y_{i}\neq {\hat{y}}_{i}}^{N}w_{i}^{\left(t\right)}/\sum_{i}^{N}w_{i}^{\left(t\right)}$ Compute weight of the t-th decision tree *α_t_*:
(9)$$\alpha_{t}=\frac{1}{2}\log\frac{\left(M-1)(1-\varepsilon_{t}\right)}{\varepsilon_{t}}$$ If *α* > 0, then Update weight of training sample $w_{i}^{\left(t+1\right)}$:
(10)$$w_{i}^{\left(t+1\right)}=\{\begin{array}{c} w_{i}^{\left(t\right)}\exp(\alpha_{t})\;if\;y_{i}\neq {\hat{y}}_{i}\\ w_{i}^{\left(t\right)}\exp(-\alpha_{t})\;otherwise \end{array}$$  else    Reject the decision tree  **End For**3. Final output: A BRF consists of N decision trees (*N ≤ T)*


**Figure 2:**
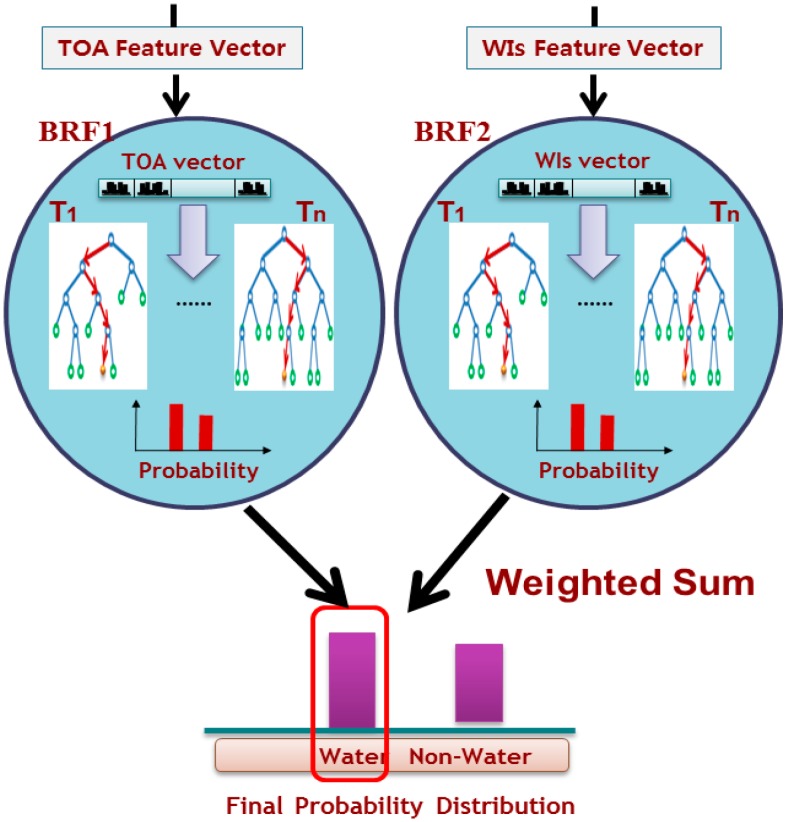
Water body classification process using the combination of two trained BRFs. The test pixel of input image is placed into the class that has the maximum posterior probability.

The two parameters of the BRF, a depth of tree (*D*) and the number of trees (*T*), are set as 20 and 120, based on the experimental results of [19]. After a set of BRFs is learned using the positive and negative training data, two feature vectors are extracted from every pixel of test data as shown in Figure 2. These vectors are used as input to the corresponding learned BRF. The probabilities of a water body class using TOA reflectance and using the WIs vector are computed by ensemble averaging of each probability distribution of all trees *L* = (*l*_1_, *l*_2_,…, *l_T_*) using Equations (11) and (12).

(11) $$P_{TOA}(C_{Water}|L)=\frac{1}{T}\sum_{t=1}^{T}P(C_{Water}|l_{t})$$

(12) $$P_{WI}(C_{Water}|L)=\frac{1}{T}\sum_{t=1}^{T}P(C_{Water}|l_{t})$$

Then, the final probability of a pixel on the water body class is estimated by weighted combination of each BRF’s probability:

(13) $$P(water)=w_{1}\cdot P_{TOA}(C_{Water}|L)+(1-w_{1})\cdot P_{WI}(C_{Water}|L)$$

The appropriate coefficient of weight *w*_1_ can be adjusted according to the characteristics of water type. We set *w*_1_ to 0.5 based on the experimental results described in Section 4. Last, if the final probability of *P(water)* exceeds a minimum threshold of 0.5, the pixel is accepted as a water body pixel. In Figure 2, the input pixel is classified into water class because the probability of water class is larger than that of non-water class.

After every pixel is classified either as a water body or background, neighboring water body pixels are merged into water regions using morphological closing. If the number of pixels of a water region is below 30 pixels after merging, it is declared as noise and removed. Proposed system is implemented using the same environment of Visual C++ language an Intel Quad-Core i7 processor PC running Windows 7 OS.

## 4. Experimental Section

Lake and river have various spectral signatures and they are hardly mapped with one or two classification values. Sometimes it’s very easy to map them just with one threshold, and any method can delineate them accurately, while sometimes classification methods cannot delineate them when river or lake has different colors according to degree of Secchi depth, turbidity, and chlorophyll-a.

Therefore, to evaluate the water body classification performance, three areas of Korea with different water types and topologies were selected:

- Area 1- Yedang artificial reservoir of Yesan city that is surrounded by agricultural areas
- Area 2- Soyangho lake of Gangwon Province that is surrounded by big mountainous areas and it also includes mountain shadows.
- Area 3- the middle Nakdonggang River of Daegu City that is surrounded by agricultural areas and mountainous areas.

**Figure 3:**
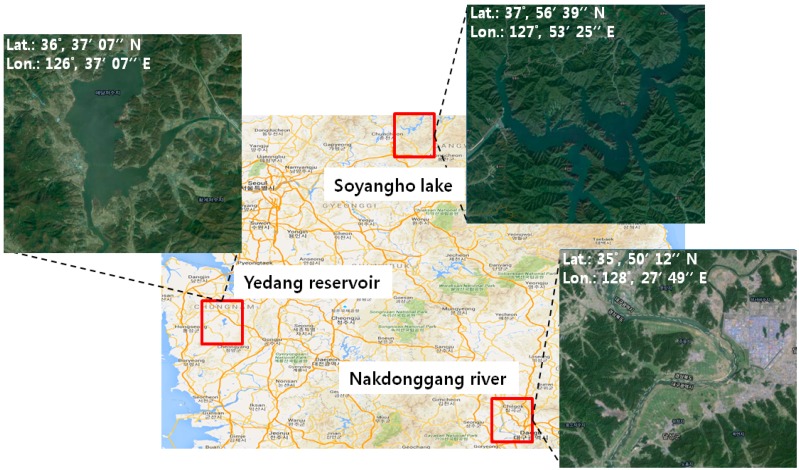
Three areas of Korea with different water types and topologies for performance evaluation. These images include many types of water features such as rivers, lakes and reservoirs.

Figure 3 shows the test images of the three areas.

In this study we used three types of Landsat sensors, *i.e.*, TM, ETM+, and OLI, in order to investigate which sensor provides the best classification result on a water body. We captured TM data on 3 July 2009, ETM+ data and OLI data on 5 July 2014, at the same three sites. Because TM data does not provide data service since 2013, most recent data of the same location was used. In addition, because the ETM+ scan line corrector failed (SLC-off) permanently, we used the “dust & scratches” filter of Adobe Photoshop to remove noises and fill the gap in the ETM+ data according to the guidelines of the United States Geological Survey (USGS) [20] before applying the proposed classification algorithm. For performance comparison, TM and ETM+ used the same TOA reflectance using the formula in [14].

The individual Landsat data set consisted of three 800 × 800 images of each area. To assess the performance of the water body classification, we asked two experts to crop the water body regions (river and lake) of test data as the ground truth using a graphic tool. To quantitatively evaluate the water body classification, we used overall accuracy (OA) and Kappa coefficient that have been generally used in related works [3,4,16,21]. The OA for each area was estimated by comparing the ground truth of water body pixels with the classified pixels obtained using the proposed system (Equation (14)) and Kappa measures the percentage of agreement between the ground truth and segmented water body pixel using Equation (15):

(14) $$OA=\frac{number\;of\;true\;positive+number\;of\;true\;negative}{All\;pixels\;in\;the\;ground\;truth}$$

(15) $$Kappa=\frac{n\sum_{k=1}^{q}n_{kk}-\sum_{k=1}^{q}n_{k+}n_{+k}}{n^{2}-\sum_{k=1}^{q}n_{k+}n_{+k}}$$

where n is the total number of pixels n the reference data, $n_{kk}$ is the total number of *i*-th class, $n_{k+}$ is the total number of pixels for the *i*-th class derived from the classified data, $n_{+k}$ is the total number of pixels for the *i*-th class derived from the reference data. q is the total number of class.

To evaluate the performance of the Landsat 8 OLI sensor, we compared its classification performance to that of the Landsat 5 TM and Landsat 7 ETM+ sensors using OA and Kappa on three test images. Table 2 shows the two components of accuracy for three different sensors. As shown in Table 2, the OLI sensor produced a better classification performance with an average OA rate of 99.90% and average Kappa of 0.9942 as compared to 99.05% and 0.9469 for the TM sensor, and 99.73% and 0.9738 for the ETM+ sensor. The main reason for higher classification rate of the Landsat 8 OLI sensor is that it provides improved SNR radiometric performance by quantizing sensed radiance into 12 bits (4096 levels) of meaningful data, rather than the 8 bits (256 levels) used by Landsat ETM+ [5]. Furthermore, OLI sensor provides narrow the spectral bands and reduces the sensitivity of the changes in the atmosphere.

The performance of the proposed classification method was then compared with two categories of state-of-the-art methods and the same approach using RFs, *i.e.*, (i) the method devised by Li *et al.* [3], which uses an Otsu threshold method (Otsu threshold) with NDWI and MNDWI and (ii) the method devised by Kalkana *et al.* [9], which uses SVM classifier (SVM); (iii) Combination of two RFs. For SVM, Gaussian radial-basis function (RBF) kernel was used to map the input vector to a higher dimensional feature space with σ = 1 because SVM with an RBF kernel performs better than other kernels. After SVM training with the same training data, if the final score of SVM exceeds a minimum threshold of 0.5, the pixel is accepted as a water body pixel. This test uses the same imageries from the only Landsat 8 OLI sensors because it showed the highest performance. Moreover, this study applied the same shadow removing relation to all comparative methods for objective performance test.

Table 3 shows that our proposed algorithm produces better water body classification performance than the other two methods. In terms of average OA, our method achieved a performance of 99.90%, which is 0.59% higher than the Otsu threshold-based method, 0.26% higher than the SVM classifier-based method, and 0.21% higher than the RF-based method. In addition, in terms of Kappa, our method achieved a performance of 0.9942, which is 0.0286 higher than the Otsu threshold-based method, 0.0123 higher than the SVM classifier-based method, and 0.0111 higher than the RF-based method.

**Table 2:** Water body classification comparison of three Landsat sensors.

Place	Sensors	OA (%)	Kappa
Area 1	TM	97.43	0.873946
Area 2	TM	99.98	0.999078
Area 3	TM	99.75	0.967846
**Average**		**99.05**	**0.946957**
Area 1	ETM+	99.76	0.987877
Area 2	ETM+	99.90	0.994649
Area 3	ETM+	99.54	0.939105
**Average**		**99.73**	**0.973877**
Area 1	OLI	99.88	0.993539
Area 2	OLI	99.90	0.994273
Area 3	OLI	99.92	0.994965
**Average**		**99.90**	**0.994259**

**Table 3:** Water body classification comparison of three algorithms.

Place	Methods	OA (%)	Kappa
Area 1	Otsu threshold	98.31	0.920227
Area 2	Otsu threshold	99.94	0.997037
Area 3	Otsu threshold	99.68	0.979753
**Average**		**99.31**	**0.965672**
Area 1	SVM	99.19	0.960726
Area 2	SVM	99.83	0.99122
Area 3	SVM	99.90	0.993993
**Average**		**99.64**	**0.98198**
Area 1	RF	99.82	0.990937
Area 2	RF	99.40	0.967351
Area 3	RF	99.86	0.991213
**Average**		**99.69**	**0.983167**
Area 1	Proposed method	99.88	0.993539
Area 2	Proposed method	99.90	0.994273
Area 3	Proposed method	99.92	0.994965
**Average**		**99.90**	**0.994259**

**Figure 4:**
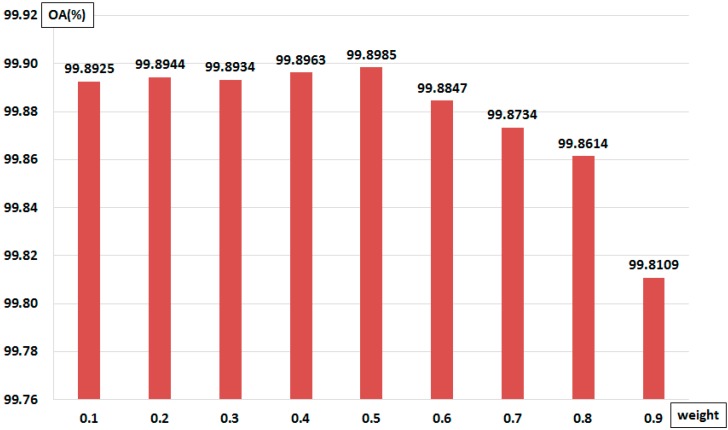
Comparison of OA performance according to the changes of weight for Equation (13).

The best classification performance was obtained for Area 3, which had an average OA of 99.92% and average Kappa of 0.9949. In contrast, Area 1 had an average OA of 99.88% and average Kappa of 0.9935. Even though the performance of SVM is similar to the proposed method, the processing speed of the proposed method is approximately 12.91 s, which is about 6 times faster than the SVM method (82.4 s) using the same testing images as shown in Figure 4. In case of RF-based method, it has somewhat lower performance than BRF-based method. From this result, we know that the segmentation accuracy can be improved by simple boosting of RF. The main reason for higher classification rate of our proposed method is that our algorithm found many potential water body pixels through individual BRF using TOA reflectance and WIs features in the first step. Our method also eliminated a large amount of false water body pixels in the second step by averaging the output probabilities of two different BRFs.

To determine the proper weights for the final probability (Equation (13)) of each feature, we compared OA performance using the same test data and proposed method while changing the value of weight. As shown in Figure 4, when $w_{1}$ was 0.5, the average OA was 99.8985%, which is better than when other coefficients were used. The experimental results show that the performance improves with the coefficient of $w_{1}$. However, when the coefficient of $w_{1}$ was greater than 0.5, the performance, in particular in terms of the OA, were gradually degraded. Therefore, $w_{1}=0.5$ was adopted as the coefficient of weight for Equation (13).

**Figure 5:**
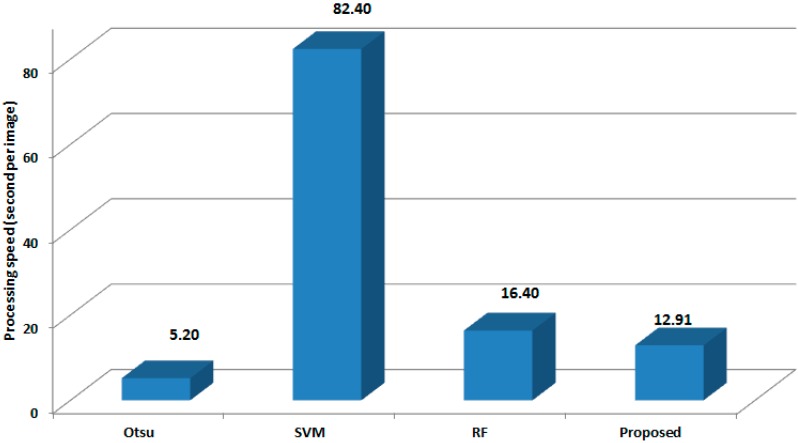
Comparison of processing speeds between proposed method and two related works.

Apart from classification disagreement, the processing speed of proposed method was compared with two three methods using the same system environment and the same testing images. As shown in Figure 5, we can certainly see that Otsu threshold reduces the processing time significantly (5.2 s per image) as compared with the proposed (12.91 s per image), RF-based method (16.4 s per image), and SVM (82.4 s per image). In a comparison of processing speed, the proposed approach shows a 7.7 s lower performance than Otsu threshold. However, classification accuracy of Otsu threshold is relatively much lower than proposed approach and accurate water body classification is important factor for water quality analysis. Even though the performance of SVM is similar to the proposed method, the processing speed of the proposed method is approximately six times faster than the SVM. From the processing speed of proposed method (12.91 s per image), we also know that the processing speed can be reduced by boosting optimal RFs. When we used the original Landsat image of 8000 × 8000 size, the classification results were almost same with the cropped test regions, but the processing time was increased by approximately 1213 s per image. In contrast, processing time for Otsu threshold was 489 s per image. One of our future works is to improve the processing speed as the similar level with Otsu threshold without losing the classification accuracy.

**Figure 6:**
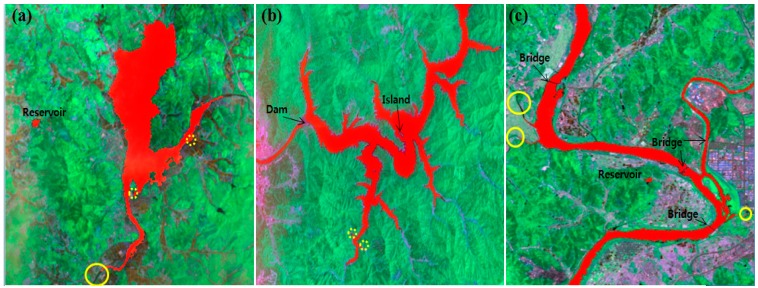
Water body classification results of three test areas, (**a**) Yedang artificial reservoir; (**b**) Soyangho lake; and (**c**) the middle Nakdonggang River of Daegu city. Classified river and lake pixels are marked in red. Miss (solid line) and false (dot line) classifications are marked in yellow circles.

Figure 6 shows the water body classification results (marked in red) obtained using four approaches at three different places. 

The classification results show that our proposed method classifies each water body correctly regardless of topology, degree of water contamination, and background. Our approach gives the best classification results, but it also produces miss or false classification results if the width of river is narrow, or if the cluttered background contains a structure similar to the river or lake, as shown in Figure 6.

## 5. Conclusions

This study proposed a robust water body classification algorithm applicable for both rivers and lakes, using TOA reflectance and WI values. For water body classification with higher accuracy, we used two types of BRF classifiers and combined the probabilities of the two BRFs into one. This study proved that the proposed method, using BRFs with two different features of the Landsat 8 OLI sensor, obtained higher classification results compared to the TM and ETM+ sensors. This was because the Landsat 8 OLI sensor provides higher SNR imagery than the other sensors. The experimental results using three datasets showed that our algorithm has enhanced classification performance compared to other state-of-the-art classification methods.

For future work, our study will first focus on reducing missing and false classification regardless of the width of river and background cluttering. Second, our study will try to improve the processing speed without losing the classification accuracy because fast processing is important component of real-time application. Finally, out study plans to focus our research on measuring water quality based on water body classification and TOA reflectance values.

## References

[1] Panda S.S., Garg V., Chaubey I. (2004). Artificial neural networks application in lake water quality estimation using satellite imagery. J. Environ. Inform..

[2] Olmanson L.G., Bauer M.E., Brezonik P.L. (2008). A 20-year Landsat water clarity census of Minnesota’s 10,000 lakes. Remote Sens. Environ..

[3] Li W., Du Z., Ling F., Zhou D., Wang H., Gui Y., Sun B., Zhang X. (2013). A comparison of land surface water mapping using the normalized difference water index from TM, ETM+ and ALI. Remote Sens..

[4] Jiang H., Feng M., Zhu Y., Lu N., Huang J., Xiao T. (2014). An automated method for extracting rivers and lakes from Landsat imagery. Remote Sens..

[5] USGS Using the USGS Landsat 8 Product. http://landsat.usgs.gov/Landsat8_Using_Product.php.

[6] Irons J.R., Dwyer J.L., Barsi J.A. (2012). The next Landsat satellite: The Landsat data continuity mission. Remote Sens. Environ..

[7] Kumar A. Landsat 8 and Landsat 7 Comparison, First Look. http://www.satpalda.com/landsat-8-and-landsat-7-comparison-first-look/.

[8] Skakun S. (2010). A neural network approach to flood mapping using satellite imagery. Comput. Inform..

[9] Kalkana K., Bayramb B., Maktava D., Sunara F. Comparison of support vector machine and object based classification methods for coastline detection. International Archives of the Photogrammetry. Proceedings of the Remote Sensing and Spatial Information Sciences (RSSIS).

[10] Neubert M., Herold H., Meinel G. Evaluation of remote sensing image segmentation quality–further results and concepts. Proceedings of the International Conference on Object-Based Image Analysis (ICOIA).

[11] Ko B.C., Kim S.H., Nam J.Y. (2011). X-ray image classification using random forests with local wavelet-based CS-local binary patterns. J. Digit. Imaging.

[12] Mcfeeters S. (1996). The use of the normalized difference water index (NDWI) in the delineation of open water features. Int. J. Remote Sens..

[13] Xu H. (2006). Modification of normalised difference water index (NDWI) to enhance open water features in remotely sensed imagery. Int. J. Remote Sens..

[14] Chandera G., Markhamb B.L., Helderc D.L. (2009). Summary of current radiometric calibration coefficients for Landsat MSS, TM, ETM+, and EO-1 ALI sensors. Remote Sens. Environ..

[15] McFeeters S.K. (2013). Using the normalized difference water index (NDWI) within a geographic information system to detect swimming pools for Mosquito Abatement: A Practical Approach. Remote Sens..

[16] Feyisa G.L., Meilby H., Fensholt R., Proud S.R. (2014). Automated water extraction index: A new technique for surface water mapping using Landsat imagery. Remote Sens. Environ..

[17] Breiman L. (2001). Random forests. Mach. Learn..

[18] Mishina Y., Tsuchiya M., Fujiyoshi H. Boosted Random Forest. Proceedings of the International Conference on Computer Vision Theory and Applications (ICCVTA).

[19] Ko B.C., Kwak J.Y., Nam J.Y. (2012). Wildfire smoke detection using temporal-spatial features and random forest classifiers. Opt. Eng..

[20] USGS-Filling the Gaps for Display. http://landsat.usgs.gov/filling_the_gaps_for_display.php#1.

[21] Foody G.M. (2002). Status of land cover classification accuracy assessment. Remote Sens. Environ..

